# Supplementary material for: The causal relationship between the human gut microbiota and pyogenic arthritis: a Mendelian randomization study
Source: Front Cell Infect Microbiol. 2024 Nov 26;14:1452480. doi: 10.3389/fcimb.2024.1452480 (PMC11629706; doi:10.3389/fcimb.2024.1452480)
Supplement: Supplementary file 1 [file DataSheet1.zip › mendelian test/ebi-a-GCST90027576.csv_forest.pdf]

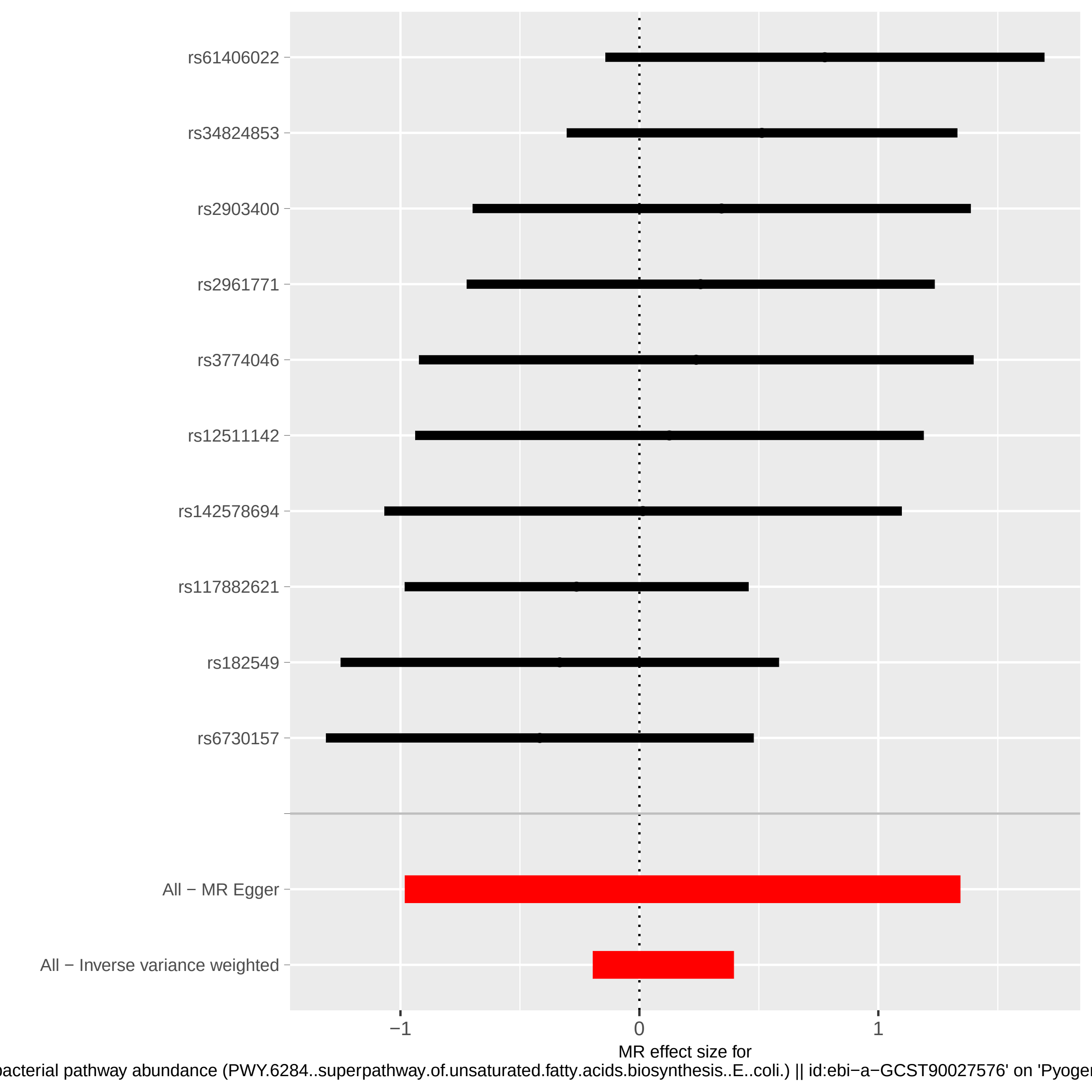
rs61406022
rs34824853
rs2903400
rs2961771
rs3774046
rs12511142
rs142578694
rs117882621
rs182549
rs6730157
All − MR Egger
All − Inverse variance weighted
−1
0
1
MR effect size for
bacterial pathway abundance (PWY.6284..superpathway.of.unsaturated.fatty.acids.biosynthesis..E..coli.) || id:ebi−a−GCST90027576' on 'Pyoger
